# Robotic Excision and Pathologic Profiling of Nonfunctioning Juxtaglomerular Cell Tumor of the Kidney: First Reported Case From the Indian Subcontinent

**DOI:** 10.7759/cureus.110539

**Published:** 2026-06-09

**Authors:** Rohit S Deshpande, Abhinav Voona, Nevitha Athikari Manamal, Akash M Shah, Yuvaraja Thyavihally

**Affiliations:** 1 Robotic Surgery and Uro-Oncology, Kokilaben Dhirubhai Ambani Hospital, Mumbai, IND; 2 Urology, Kokilaben Dhirubhai Ambani Hospital, Mumbai, IND; 3 Pathology, Kokilaben Dhirubhai Ambani Hospital, Mumbai, IND

**Keywords:** immunohistochemistry, juxtaglomerular cell tumor, nephron-sparing surgery, robotic partial nephrectomy, small renal mass

## Abstract

Juxtaglomerular cell tumor (JGCT), also known as reninoma, is an exceptionally rare benign renal neoplasm arising from modified smooth muscle cells of the afferent arteriole of the juxtaglomerular apparatus. The tumor classically presents in young patients with secondary hypertension due to autonomous renin secretion, hypokalemia, and hyperaldosteronism.

A 56-year-old asymptomatic female with known hypothyroidism was incidentally detected to have a right renal mass on a routine health check-up. Contrast-enhanced computed tomography (CECT) confirmed a 2.7 × 2.5 cm homogeneously enhancing medullary lesion in the inter and lower polar region of the right kidney with mass effect and calyceal splaying. She underwent robot-assisted right partial nephrectomy. Histopathology confirmed a juxtaglomerular cell tumor, limited to the kidney, with uninvolved margins. Immunohistochemistry showed diffuse positivity for vimentin, smooth muscle actin (SMA), caldesmon, and CD34.

This case is atypical in its presentation - an older, asymptomatic, post-menopausal female without the classic triad of hypertension, hypokalemia, and elevated renin levels. Nephron-sparing surgery is the treatment of choice whenever feasible. A high index of suspicion and a characteristic immunohistochemistry (IHC) profile are essential for definitive diagnosis.

## Introduction

Juxtaglomerular cell tumor (JGCT), first described by Robertson et al. in 1967 [1] and formally named by Kihara et al. in 1968 [2], is an exceptionally rare benign renal neoplasm. Also referred to as reninoma, it originates from the modified smooth muscle cells (myoepithelial cells) of the afferent arteriole of the juxtaglomerular apparatus - the specialized renin-secreting unit of the kidney.

The classic clinical presentation is a triad of secondary hypertension due to autonomous renin secretion leading to hyperaldosteronism, hypokalaemia, and markedly elevated plasma renin levels. It predominantly affects young women in the second to third decade of life [1,2]. The lesion is typically small (<3 cm), well-circumscribed, unilateral, and confined to the renal cortex or medulla.

Radiologically, JGCT can closely mimic renal cell carcinoma (RCC), angiomyolipoma (AML), or other renal tumours, making preoperative diagnosis challenging. The definitive diagnosis rests on histopathology and immunohistochemistry (IHC), with the tumor characteristically expressing Vimentin, CD34, smooth muscle actin (SMA), and caldesmon while being negative for epithelial (PAX-8, CK) and neuroendocrine markers [3].

Surgical excision - ideally nephron-sparing - is curative. We report an unusual case of an incidentally detected JGCT in an older asymptomatic female, successfully managed by robotic-assisted partial nephrectomy at a tertiary uro-oncology center. Fewer than 150 cases have been reported in the world literature [3]. To the best of our knowledge, this is the sixth case of asymptomatic JGCT in the world and the first to be reported from the Indian subcontinent.

## Case presentation

A 56-year-old post-menopausal female with well-controlled hypothyroidism presented to us with a renal mass, which had been incidentally detected on a routine health check-up. She had no prior surgical history.

**Figure 1:**
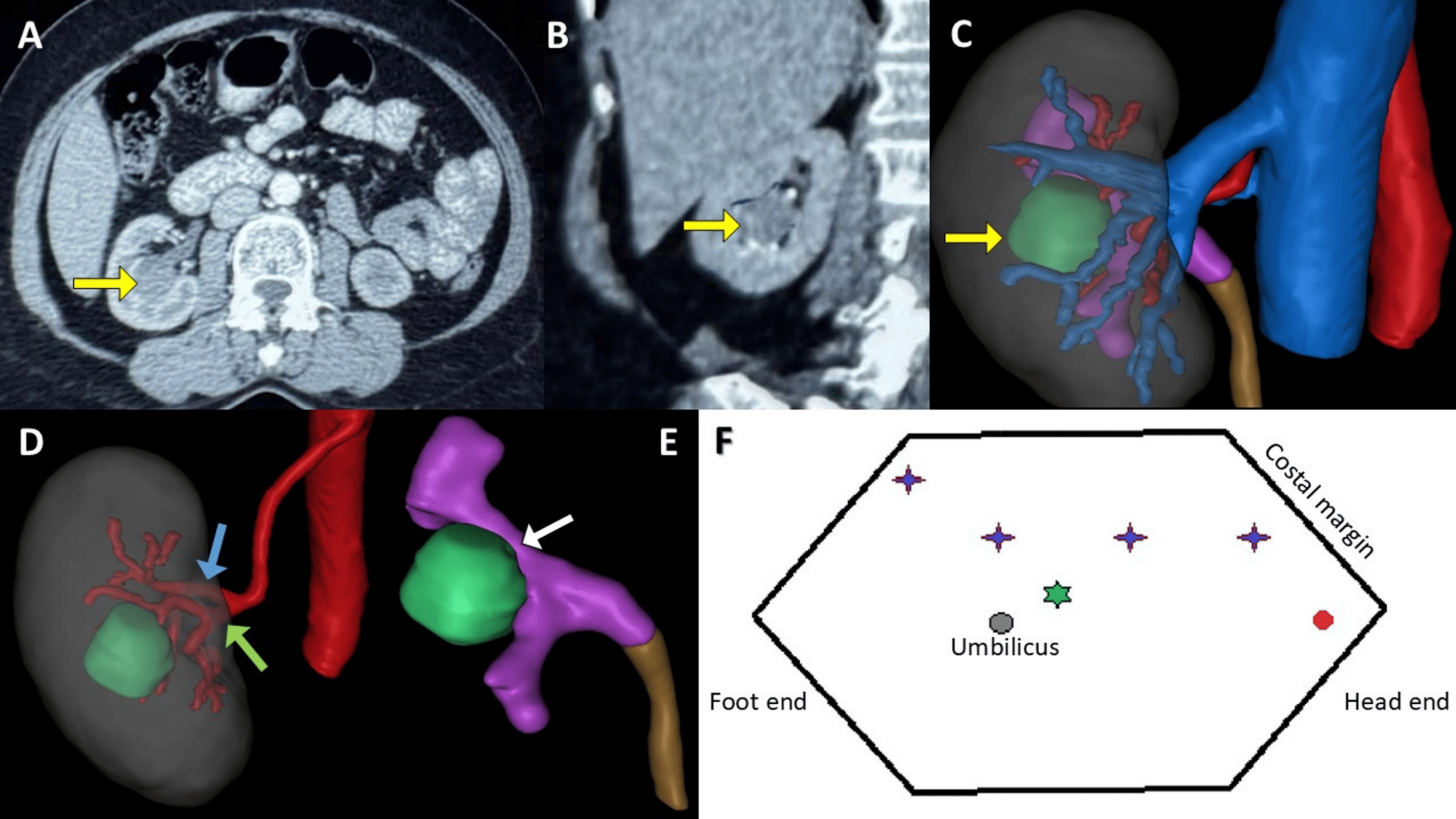
Pre-operative planning with CECT abdomen and three-dimensional reconstruction A) Axial view of nephrographic phase of CECT, depicting the renal mass (marked with yellow arrow); B) Coronal view of nephrographic phase of CECT, depicting the renal mass (marked with yellow arrow); C) Three-dimensional reconstruction of the right kidney, demonstrating the completely endophytic nature of the renal mass (marked with yellow arrow); D) Three-dimensional reconstruction of the vascular system, demonstrating the anterior (marked with green arrow) and posterior (marked with blue arrow) divisions of the right renal artery; E) Three-dimensional reconstruction of the collecting system, demonstrating the nearness (marked with white arrow) of the tumor to the pelvicalyceal system (shown in purple color); F) Port sites (with patient in left lateral decubitus): red circle depicts the 5 mm liver-retraction port site, blue stars depict Da Vinci port sites (8 mm each), green star depicts assistant port site (12 mm)

Ultrasound showed a 2.2 × 2.1 × 1.5 cm solid-cystic right renal medullary mass, with few internal septa and internal vascularity. Contrast-enhanced computed tomography (CECT) characteristically demonstrated a 2.7 × 2.5 cm well-defined homogeneous post-contrast enhancing medullary lesion, inter and lower polar right kidney, calyceal splaying; no vascular invasion - features that, while raising suspicion of a neoplastic process, did not conclusively differentiate JGCT from a small RCC (Figure 1).

**Figure 2:**
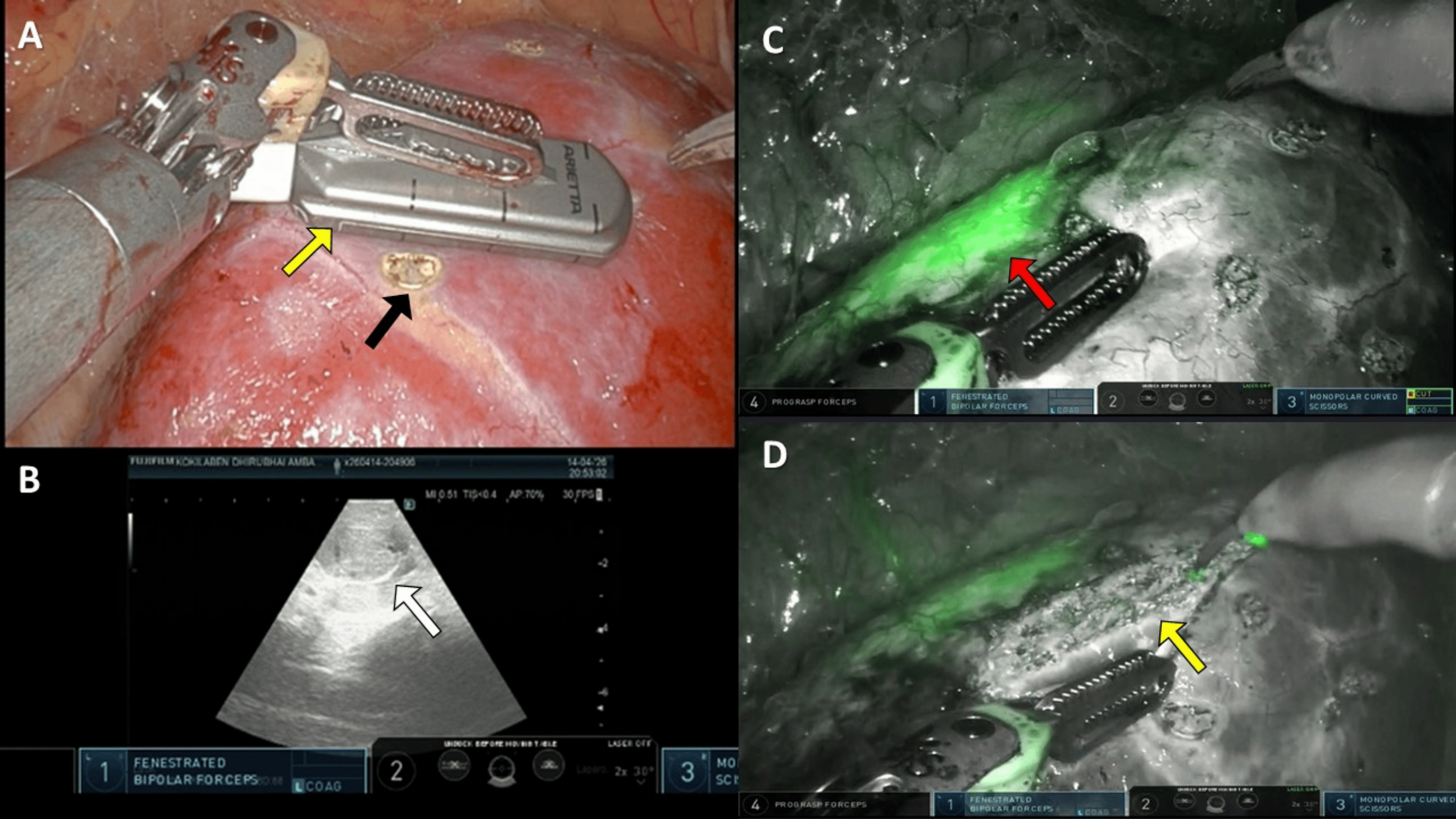
Intraoperative ultrasound and indocyanine green (ICG) fluorescence A) Intra-operative ultrasound probe (marked with yellow arrow) used for surface marking of the tumor (depicted with black arrow) using robotic scissors; B) Corresponding ultrasound image depicting the circumscribed tumor outline (shown with white arrow); C) ICG fluorescence (perfused area depicted with red arrow) used after clamping the anterior division of the right renal artery; helped to demarcate territorial distribution of arterial divisions; D) Incision being taken on the kidney, along Brodel's avascular plane (depicted by the yellow arrow)

**Figure 3:**
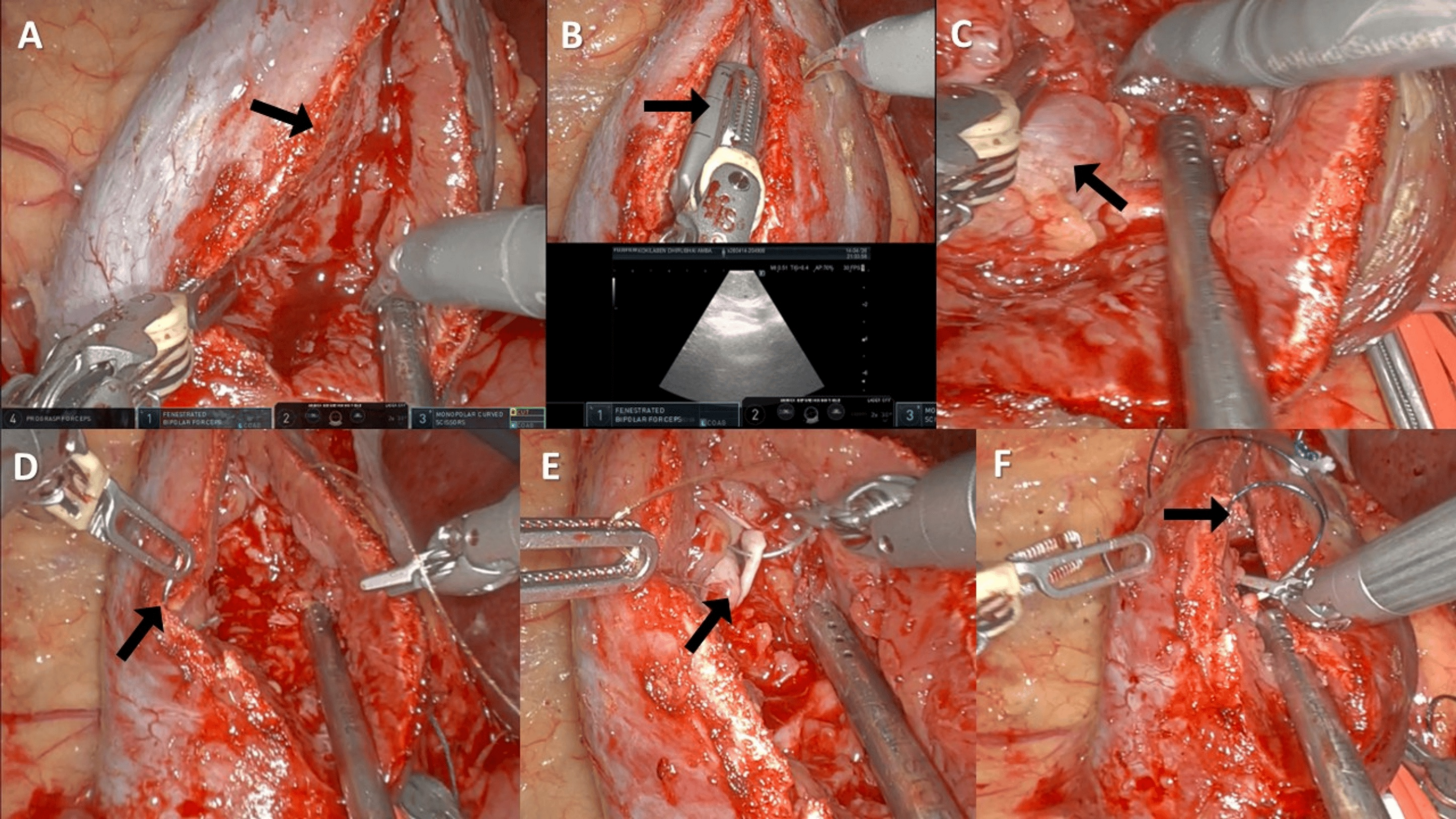
Intraoperative steps A) Nephrotomy (shown with black arrow); B) Intraoperative ultrasound (shown with black arrow) used intra-renally to delineate tumoral boundaries, with corresponding ultrasound image; C) Tumor (surface shown with black arrow) enucleo-resection in progress; D) Internal renorrhaphy done with Stratafix 3-0 suture (Johnson & Johnson MedTech, New Brunswick, New Jersey) (marked with black arrow); E)- Closure of the pelvicalyceal system (shown with black arrow) incorporated in the internal renorrhaphy, with Stratafix 3-0 suture; F) External renorrhaphy done with Stratafix 2-0 suture (Johnson & Johnson MedTech, New Brunswick, New Jersey) (shown with black arrow)

Robot-assisted right partial nephrectomy with DJ stenting was then performed. The patient was placed in the left lateral decubitus position; standard four trocars and one 12 mm trocar were inserted, and the robot was docked. Hilar dissection identified two renal veins and one artery. Gerota's fascia was dissected, and the endophytic tumor was identified using intraoperative ultrasound (Figure 2). The anterior and posterior renal arteries were clamped using bulldog clamps. Wide excision of the tumor was performed, followed by frozen section from the base - reported negative. Inner renorrhaphy was performed with Stratafix 3-0 (Johnson & Johnson MedTech, New Brunswick, New Jersey), followed by outer renorrhaphy with Stratafix 2-0 (Johnson & Johnson MedTech, New Brunswick, New Jersey) (Figure 3). Console time was ~104 minutes, and warm ischemia time was ~35 minutes. The postoperative course was largely uneventful. The patient was discharged on post-operative day four. The stent was removed after one month, and the serum creatinine was normal at follow-up.

On microscopy, the tumor was well-circumscribed and composed of sheets of round to polygonal cells with eosinophilic cytoplasm and distinct cell borders. Interspersed vascularity was prominent. No mitotic activity or nuclear atypia was identified.

**Figure 4:**
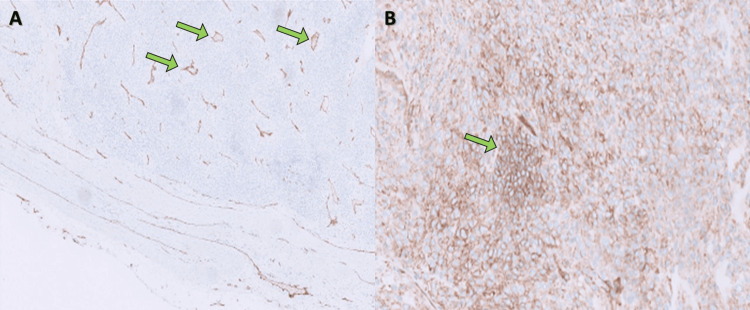
Immunohistochemistry profiling A) Immunohistochemistry (10x magnification) showing vascularity highlighted by CD34 (marked with green arrow); B) Immunohistochemistry (10x magnification) for CD34 showing positivity in tumor cells (marked with green arrow)

On immunohistochemistry (IHC), tumor cells were diffusely positive for vimentin, smooth muscle actin (SMA), caldesmon, and CD34 (Figure 4). They were negative for PAX-8, CK7, CK20, Gata-3, S-100, synaptophysin, chromogranin A, CD117, CD31, CK, epithelial membrane antigen (EMA), and β-catenin. No clear cell or papillary areas were identified. 

## Discussion

The majority of juxtaglomerular cell tumors are benign. The classic clinico-pathological profile - young female, secondary hypertension, hypokalaemia, and elevated plasma renin - is well-established, and surgical cure is invariably associated with resolution of hypertension [3]. To the best of our knowledge, no cases of metastatic JGCT have been reported to date [4]. Fewer than 150 cases of JGCT have been described in the global literature since its first description in 1967 [3,5]. Large size, necrosis, and increased mitoses are considered atypical features warranting close follow-up [6]. Rare vascular invasion has been reported [7]. Our case is atypical in several important aspects.

First, the patient was 56 years old - considerably older than the typical second-to-third decade presentation. Second, she was entirely asymptomatic, without the signature secondary hypertension or hypokalaemia alkalosis. Third, the lesion was detected incidentally, not as a work-up for refractory hypertension. This underscores an important emerging recognition: JGCT can present without its classic triad, especially in older patients, and may be entirely occult clinically. As per the available medical literature, this is the sixth case of asymptomatic JGCT in the world [8-12], and to our knowledge, the first to be reported from the Indian subcontinent*. *Our case can be classified as belonging to non-functioning variants of JGCT [12].

Radiologically, JGCT typically appears as a small (<3 cm), well-defined, hypovascular to moderately enhancing solid mass, often in the cortex. In this patient, the CECT showed a 2.7 × 2.5 cm homogeneously enhancing medullary lesion - a pattern easily mistaken for a small clear cell RCC (cT1a), AML with minimal fat, or a metanephric adenoma. The absence of fat density excluded lipid-rich AML; the medullary location and morphology raised suspicion, but pre-operative tissue diagnosis was not pursued, as is standard practice for small solid renal masses amenable to nephron-sparing resection.

The IHC profile is pathognomonic. CD34 and SMA positivity, along with caldesmon expression, reflect the myoepithelial (modified smooth muscle) lineage of the juxtaglomerular cells. PAX-8 negativity excludes a renal epithelial/RCC origin; CD117 negativity excludes gastrointestinal stromal tumor (GIST); neuroendocrine marker negativity (chromogranin, synaptophysin) excludes carcinoid. The absence of nuclear atypia, mitoses, and necrosis is consistent with its universally benign behavior. 

Nephron-sparing surgery - whether open, laparoscopic, or robotic - is the treatment of choice whenever feasible [13] and is curative. The robotic approach offered the advantages of enhanced 3D visualization, tremor-free dissection, and articulated instrumentation within a confined retroperitoneal space, facilitating precise tumor excision with minimal warm ischemia. Intraoperative frozen section from the resection margin was a useful adjunct to confirm adequacy of excision, as was done in this case (margin confirmed negative).

Postoperative surveillance strategy for JGCT is not standardized, given its extreme rarity. Given the confirmed negative margins in this case, the long-term prognosis is excellent. 

## Conclusions

Juxtaglomerular cell tumor is a rare but important differential diagnosis for small renal masses, even in the absence of the classic clinical triad of secondary hypertension, hypokalemia, and elevated renin. This case underscores the indispensable role of histopathology and immunohistochemistry in establishing the diagnosis. Nephron-sparing surgery, including robotic partial nephrectomy when technically feasible, is an appropriate curative approach. Awareness of this entity among urologists, radiologists, and pathologists is critical to avoid misclassification and ensure appropriate management.

## References

[1] Robertson PW, Klidjian A, Harding LK, Walters G, Lee MR, Robb-Smith AH (1967). Hypertension due to a renin-secreting renal tumour. Am J Med.

[2] Kihara I, Kitamura S, Hoshino T, Seida H, Watanabe T (1968). A hitherto unreported vascular tumor of the kidney: a proposal of "juxtaglomerular cell tumor". Acta Pathol Jpn.

[3] Corvol P, Pinet F, Galen FX (1988). Seven lessons from seven renin secreting tumors. Kidney Int Suppl.

[4] Méndez GP, Klock C, Nosé V (2011). Juxtaglomerular cell tumor of the kidney: case report and differential diagnosis with emphasis on pathologic and cytopathologic features. Int J Surg Pathol.

[5] Kuroda N, Gotoda H, Ohe C (2011). Review of juxtaglomerular cell tumor with focus on pathobiological aspect. Diagn Pathol.

[6] Munakata S, Tomiyama E, Takayama H (2018). Case report of atypical juxtaglomerular cell tumor. Case Rep Pathol.

[7] Beaudoin J, Périgny M, Têtu B, Lebel M (2008). A patient with a juxtaglomerular cell tumor with histological vascular invasion. Nat Clin Pract Nephrol.

[8] Endoh Y, Motoyama T, Hayami S, Kihara I (1997). Juxtaglomerular cell tumor of the kidney: report of a non-functioning variant. Pathol Int.

[9] Dong J, Xu W, Ji Z (2020). Case report: a nonfunctioning juxtaglomerular cell tumor mimicking renal cell carcinoma. Medicine (Baltimore).

[10] Sakata R, Shimoyamada H, Yanagisawa M (2013). Nonfunctioning juxtaglomerular cell tumor. Case Rep Pathol.

[11] Takahashi T, Miura T, Sue A (1976). A case of juxtaglomerular cell tumor diagnosed preoperatively. Nephron.

[12] Dong D, Li H, Yan W, Xu W (2010). Juxtaglomerular cell tumor of the kidney--a new classification scheme. Urol Oncol.

[13] Mete UK, Niranjan J, Kusum J, Rajesh LS, Goswami AK, Sharma SK (2003). Reninoma treated with nephron-sparing surgery. Urology.

